# Iatrogenic Paradoxical Stroke in a Patient With Catheter-Associated Thrombosis and Systemic-to-Pulmonary Venous Shunt

**DOI:** 10.1177/2324709618813175

**Published:** 2018-11-15

**Authors:** Lucas J. Policastro, Kristaq Koci

**Affiliations:** 1 SUNY Downstate Medical Center, Brooklyn, NY, USA

**Keywords:** systemic-to-pulmonary venous shunt, paradoxical embolism, paradoxical stroke, right-to-left shunt, central venous access device

## Abstract

Paradoxical embolism occurs when thrombotic material traverses a right-to-left shunt. We describe the first case of paradoxical stroke resulting from manipulation of a disused chemotherapy port. Contrast studies revealed that the mechanism was systemic-to-pulmonary venous shunt, in which systemic veins drain into the left atrium via collaterals. Chronically thrombosed central venous catheters may result in venous stenosis and shunt formation, exposing patients to risks of paradoxical stroke, acute coronary syndrome, hypoxemia, and other complications. This case highlights the life-threatening complications that may result from neglect of an implantable central venous catheter. Preventative measures are to promptly recognize and treat catheter-related thrombosis and to remove unneeded catheters.

## Introduction

Paradoxical embolism refers to arterial embolism resulting from thrombotic material of venous origin. Catheter-associated thrombosis is a frequent complication of long-term indwelling central venous catheters (CVCs)^1^ and may result in right-to-left shunt formation. Systemic-to-pulmonary venous shunt (SPVS) is a rarely reported type of right-to-left shunt, in which systemic veins drain into the pulmonary veins or left atrium.^2,3^

## Case Report

A man in his 60s with prostate cancer metastatic to bone and end-stage renal disease was brought to the hospital by his wife due to several days of reduced appetite and inability to ambulate independently. He had a history of strokes suffered 2 years ago and 10 months ago, with residual aphasia. Five months ago, he was admitted for sepsis associated with his tunneled dialysis catheter, with catheter tip and blood cultures having grown *Enterobacter cloacae*.

On presentation, he was febrile to 104°F, normotensive, had a heart rate of 134 beats per minute, and oxygen saturation was 95%. He possessed a right chest dialysis catheter tunneled to the right internal jugular vein, and a left chest subcutaneous chemotherapy port entering the left subclavian vein. Neurologic examination confirmed expressive aphasia. Antibiotics were started, and he was admitted to the hospital for probable catheter-associated sepsis.

**Figure 1.**
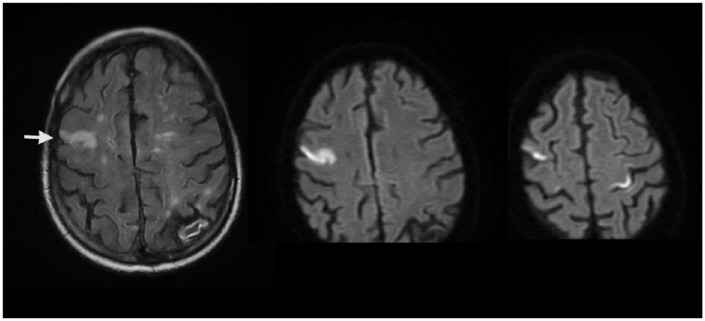
Magnetic resonance imaging demonstrating acute ischemic stroke. Left, T2 FLAIR image showing a high signal lesion in the right frontal lobe. Center and right, diffusion-weighted images showing bilateral positive gyriform signals in the right and left posterior frontal lobes.

After the patient’s arrival at the medical floor, an intern embarked to obtain differential blood cultures. A large-bore needle was inserted into the chemotherapy port and gentle negative pressure was applied to the syringe, with no return of blood. The attempt was aborted and the intern moved to the right side to inspect the dialysis catheter; however, the patient was found to have lost consciousness. Vital signs were normal, and examination revealed torticollis and gaze deviation to the right; a stroke code was promptly called. After evaluation by the neurologist, the patient was transported for head computed tomography (CT). Approximately 20 minutes later, the patient regained consciousness while on the CT table. The scan showed no acute changes. Ultimately, the event was suspicious more for complex seizure than stroke; therefore, thrombolytic treatment was not given. Nonetheless, follow-up brain magnetic resonance imaging revealed new ischemia in the right and left frontal lobes (Figure 1). Neurological examination progressed to prominent right-sided weakness, which was not present on admission.

Blood cultures grew *Klebsiella pneumoniae*. To prevent recurrent bacteremia, the tunneled dialysis catheter was removed at bedside on hospital day 2, and the patient was booked for removal of the chemotherapy port under general anesthesia. He recovered readily from sepsis.

**Figure 2.**
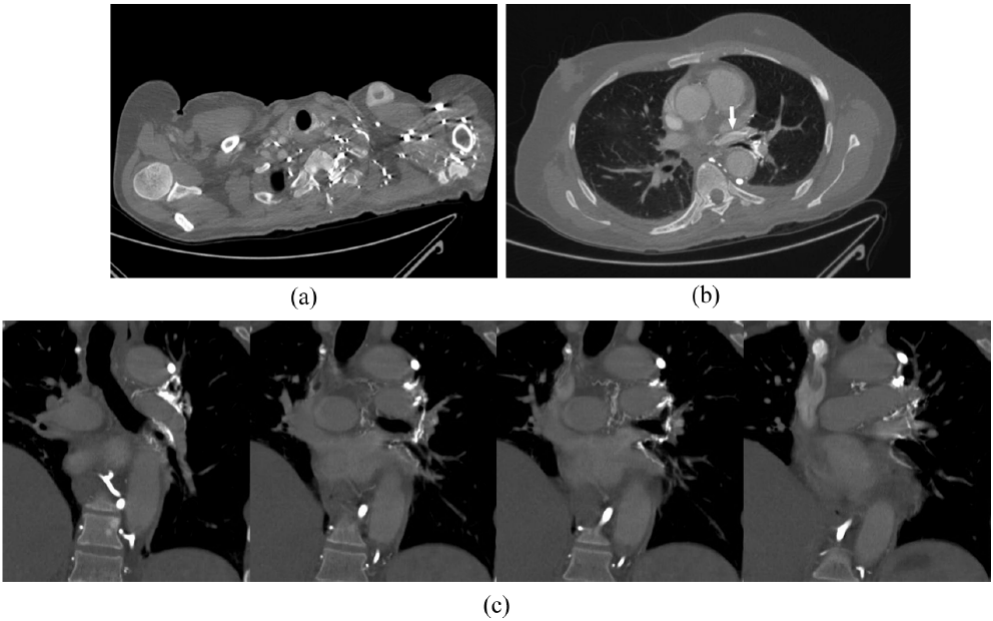
Chest computed tomography angiogram conducted under pulmonary embolism protocol. Contrast was injected into a left upper extremity vein. (a) Venous collaterals are well-developed as a result of left brachiocephalic vein stenosis. (b) Contrast is seen within the left superior pulmonary vein (arrow). (c) Four coronal slices are shown, demonstrating collateralization of the left bronchial venous plexus as well as enhancement of the left pulmonary vein.

**Figure 3.**
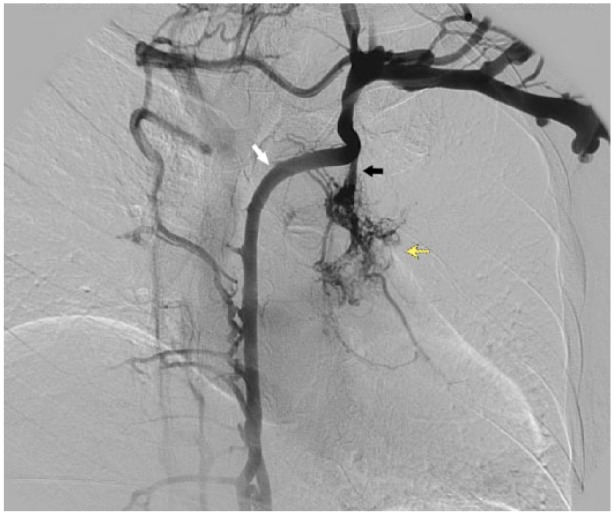
Digital subtraction venogram demonstrating collateral flow from the left upper extremity. The accessory hemiazygos vein (white arrow) has become a prominent collateral of the left subclavian. Flow is reversed within the left bronchial veins (black arrow), seen receiving blood from the accessory hemiazygos vein and/or left superior intercostal vein. As a result, the left bronchial venous plexus is filled with contrast (yellow-dashed arrow). This plexus drains to the pulmonary veins.

A chart review was conducted. The chemotherapy port had been implanted several years ago and was no longer in use. During the patient’s admission for stroke 10 months ago, contrast echocardiography had revealed a right-to-left shunt, with contrast appearing in the left atrium 2 to 3 beats prior to the right atrium. No atrial septal defect was seen on transesophageal echocardiogram. The technologist noted that the shunt was visible with contrast injected into the left arm, but not the right. This finding was followed-up with a CT venogram of the chest, which failed to identify the source of shunt. At this point, the diagnostic inquiry ended. We sought a radiologist’s review of the CT scan and ascertained that it was nondiagnostic due to incorrect contrast phasing; therefore, we repeated the scan under a pulmonary embolism protocol. This CT angiogram showed left brachiocephalic vein stenosis related to the catheter tip, partial superior vena cava thrombus, and extensive venous collateralization via intercostal, mediastinal, and azygos veins (Figure 2a). There was increased density in the left superior pulmonary vein (Figure 2b) suggestive of SPVS. Coronal images revealed filling of the left bronchial venous plexus (Figure 2c), which drains into the pulmonary veins.^4^ Interventional radiology was consulted to perform a traditional venogram (Figure 3).

A risk-benefit analysis was performed, weighing the risk of removing the chemotherapy port to prevent further bacteremia against keeping the port due to the possibility of precipitating further emboli during port extraction. After discussion with the surgery service, it was decided to forgo removal of the port. The patient was bridged with heparin to long-term warfarin therapy. He was referred to the infectious disease clinic for close monitoring, and discharged to subacute rehabilitation.

## Discussion

In this case, bilateral ischemic stroke was provoked by an attempt to draw blood from a disused implantable central venous access port. This is the first report of paradoxical thromboembolism caused by manipulation of a CVC. It is also a rare case of paradoxical embolism resulting from SPVS, previously reported.^2,5-7^ Before this incident, our patient had suffered 2 strokes, which left him aphasic, possibly also attributable to paradoxical embolism.

**Figure 4.**
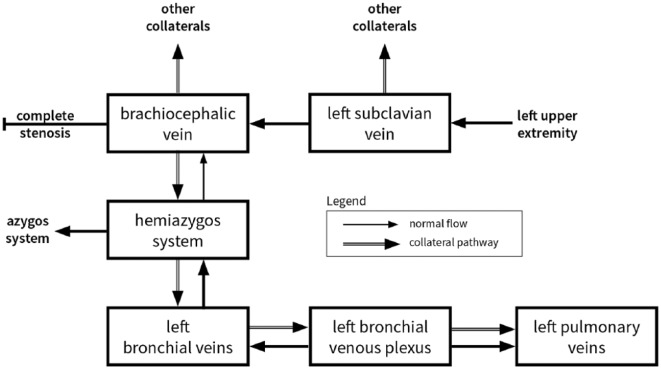
A schematic of the collateral venous flow in systemic-to-pulmonary venous shunt via the left bronchial veins.

Head CT showed no intracranial air; therefore, we suspect thromboembolism rather than air embolism. Speculatively, negative pressure from the syringe was transmitted through the catheter, causing motion within the organized clot of the left brachiocephalic vein. This motion engendered the formation or dislodgement of thrombotic material distal to the stenosed region of vein. As evident in Figure 3, there was strong collateral flow to the accessory hemiazygos vein, causing reversal of flow into the left bronchial veins, normally protected by valves. Further backflow can be seen into the bronchial venous plexus, which drains to the pulmonary veins, thus establishing a right-to-left shunt and the route by which the thrombi traveled (see Figure 4).

The American Society of Clinical Oncology guidelines note that “prolonged retention of an unneeded CVC can lead to significant problems associated with thrombosis and fibrosis.”^8^ Most cases of upper extremity deep vein thrombosis (UE DVT) are secondary to CVC use, and its chief complication is post-thrombotic syndrome.^9^ Reported rates of pulmonary embolism from UE DVT have varied widely over time, more recently ranging from 1% to 9% with low mortality.^10,11^ Current American College of Chest Physicians guidelines recommend anticoagulation for acute UE DVT.^12^ Evidence including the WARP (Warfarin thromboprophylaxis in cancer patients with CVCs) trial has not suggested a net benefit from prophylactic anticoagulation.^1^ When thrombosis does not respond to fibrinolytic or anticoagulation therapy, the American Society of Clinical Oncology recommends catheter removal.^8^ This case demonstrates the potential complications of a “forgotten” implantable port: from thrombosis, to stenosis, shunt, and finally paradoxical embolism. Other than stroke, paradoxical embolism may cause acute coronary syndrome and cerebral abscesses. Our patient had a mildly decreased oxygen saturation of 95%; however, SPVS often presents with dyspnea secondary to hypoxemia.^2^

As noted by Gilkeson et al, increased long-term use of central venous access devices has placed a large population at risk for venous stenosis.^2^ SPVS may be an underrecognized condition in cases of central venous obstruction.^13^
